# Optical coherence tomography-based contact indentation for diaphragm mechanics in a mouse model of transforming growth factor alpha induced lung disease

**DOI:** 10.1038/s41598-017-01431-x

**Published:** 2017-05-04

**Authors:** Kimberley C. W. Wang, Chrissie J. Astell, Philip Wijesinghe, Alexander N. Larcombe, Gavin J. Pinniger, Graeme R. Zosky, Brendan F. Kennedy, Luke J. Berry, David D. Sampson, Alan L. James, Timothy D. Le Cras, Peter B. Noble

**Affiliations:** 1 Telethon Kids Institute, The University of Western Australia, Subiaco, Western Australia Australia; 2 School of Anatomy, Physiology and Human Biology, The University of Western Australia, Perth, Western Australia Australia; 3 Optical+Biomedical Engineering Laboratory, School of Electrical, Electronic & Computer Engineering, The University of Western Australia, Perth, Western Australia Australia; 4 BRITElab, Harry Perkins Institute of Medical Research QEII Medical Centre, Crawley, Western Australia Australia; 5 School of Public Health, Curtin University, Perth, Western Australia Australia; 6 University of Tasmania, Hobart, Tasmania Australia; 7 School of Electrical, Electronic & Computer Engineering, The University of Western Australia, Perth, Western Australia Australia; 8 Centre for Microscopy, Characterisation & Analysis, The University of Western Australia, Perth, Western Australia Australia; 9 Sir Charles Gairdner Hospital, Nedlands, Western Australia Australia; 10 Cincinnati Children’s Hospital Medical Center, Cincinnati, Ohio USA; 11 Centre for Neonatal Research and Education, School of Paediatrics and Child Health, The University of Western Australia, Perth, Western Australia Australia

## Abstract

This study tested the utility of optical coherence tomography (OCT)-based indentation to assess mechanical properties of respiratory tissues in disease. Using OCT-based indentation, the elastic modulus of mouse diaphragm was measured from changes in diaphragm thickness in response to an applied force provided by an indenter. We used a transgenic mouse model of chronic lung disease induced by the overexpression of transforming growth factor-alpha (TGF-α), established by the presence of pleural and peribronchial fibrosis and impaired lung mechanics determined by the forced oscillation technique and plethysmography. Diaphragm elastic modulus assessed by OCT-based indentation was reduced by TGF-α at both left and right lateral locations (*p* < 0.05). Diaphragm elastic modulus at left and right lateral locations were correlated within mice (r = 0.67, *p* < 0.01) suggesting that measurements were representative of tissue beyond the indenter field. Co-localised images of diaphragm after TGF-α overexpression revealed a layered fibrotic appearance. Maximum diaphragm force in conventional organ bath studies was also reduced by TGF-α overexpression (*p* < 0.01). Results show that OCT-based indentation provided clear delineation of diseased diaphragm, and together with organ bath assessment, provides new evidence suggesting that TGF-α overexpression produces impairment in diaphragm function and, therefore, an increase in the work of breathing in chronic lung disease.

## Introduction

Optical coherence tomography (OCT) is an interferometric imaging technique that forms volumetric images of tissue structure based on contrast generated by the back-scattering of light waves^1^. In the respiratory field, an increasing number of new applications for OCT are emerging^2^. Various endoscopic, needle and bench top OCT systems have been used to measure lumen dimensions in small^3^, large^4, 5^ and upper airways^6^, to assess wall morphology and thickness^4, 7^, and to visualise individual alveoli^8^. However, one feature of OCT in the respiratory system that could provide significant clinical application has yet to be fully exploited – assessment of respiratory tissue mechanics.

While the primary application of imaging techniques such as OCT is to provide information on tissue structure, the approach may be extended to the assessment of tissue mechanics (e.g., elastic modulus) by measuring deformation of structure in response to an applied force^9, 10^. Indentation assessed by OCT, hereafter referred to as ‘OCT-based indentation’^11–14^ measures tissue deformation and structure, providing complementary information pertaining to organ function. This promising technology provides an opportunity to better understand the functional consequences of disease-related changes in tissue structure and mechanics, not possible using more standard techniques in respiratory mechanics^15^.

We used OCT-based indentation to characterise diaphragm dysfunction in a transgenic mouse model of transforming growth factor-alpha (TGF-α)-induced lung disease. Overexpression of TGF-α in mice has been shown to produce lung and pleural fibrosis and impair respiratory mechanics^16, 17^. We further questioned whether the pathology would extend to the diaphragm and alter its function. Chronic lung disease produced by TGF-α was initially established by histology and changes in lung mechanics assessed by the forced oscillation technique (FOT) and plethysmography, and the proposed changes in diaphragm function were demonstrated by the assessment of contractile force in organ bath chambers. The aim of the study was to determine whether OCT-based indentation could delineate changes in diaphragm mechanics in a mouse model of chronic lung disease.

## Results

**Figure 1:**
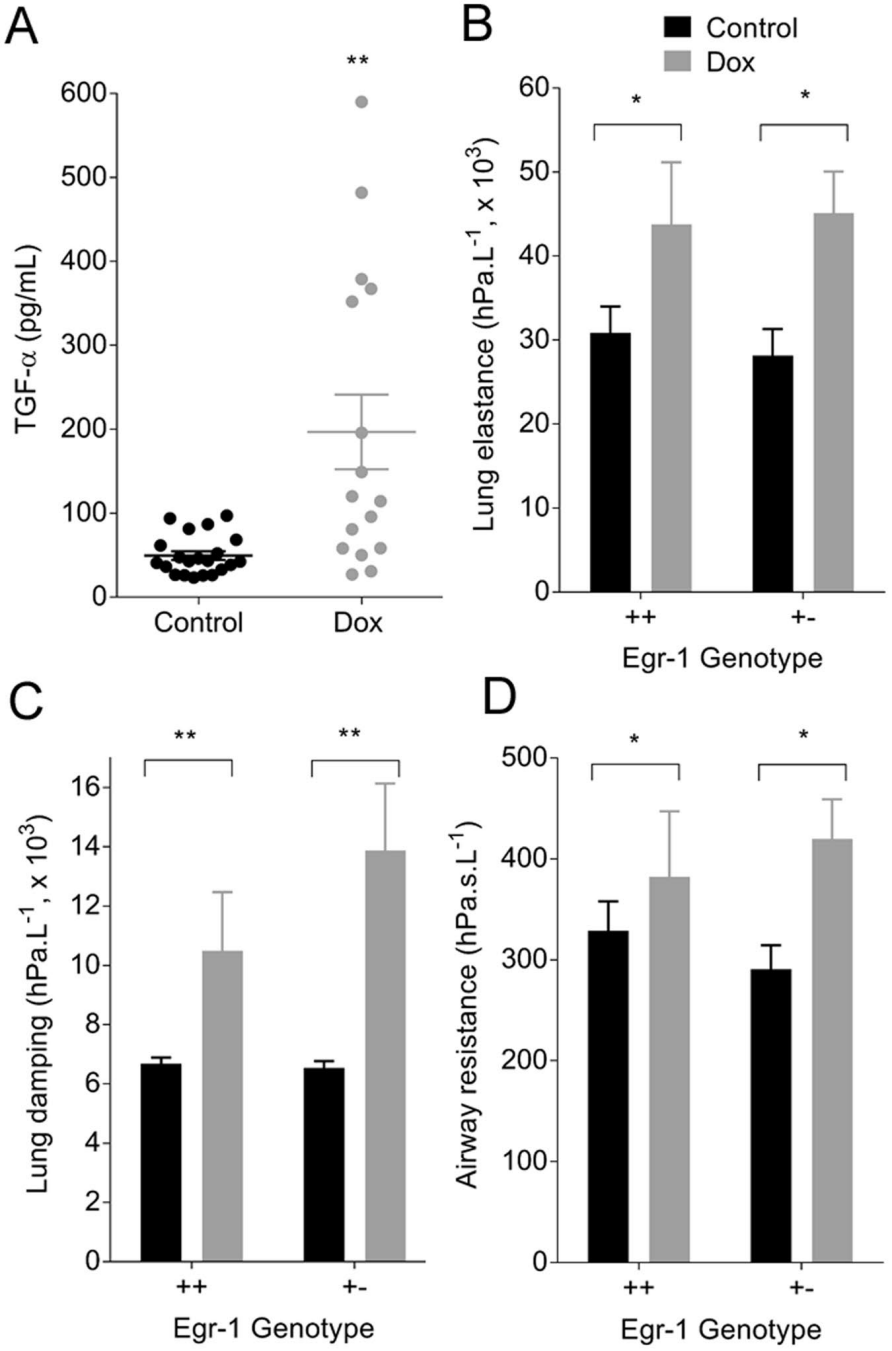
Dox induced TGF-α overexpression (**A**, Control n = 21; Dox n = 16; **p < 0.01) and increased lung elastance (**B**, *p < 0.05), lung damping (**C**, ***p < 0.01) and airway resistance (**D**, *p < 0.05) compared with the Control group. There was no effect of genotype. Data are mean ± SE; Egr-1++ Control n = 7, Dox n = 7; Egr-1+− Control n = 7, Dox n = 12.

As fully described in the Methods, lung disease was induced by TGF-α overexpression in transgenic mice, triggered by doxycycline (Dox) in the diet. Levels of TGF-α in lung homogenates were increased by Dox (Fig. 1A). Animal characteristics are provided in Table 1. In the FOT study, mice treated with Dox were marginally smaller than the Control group which contained a greater proportion of males. There were no differences between Dox and Control groups in the other studies.

**Table 1:** Group characteristics.

	Control		Doxycycline	
	Sex (M:F)	Mass (g)	Sex (M:F)	Mass (g)
FOT	12:6	21.9 ± 0.8	9:11	19.5 ± 0.7*
OCT	2:6	20.8 ± 1.0	5:5	22.2 ± 0.8
Organ bath	4:5	21.8 ± 1.1	6:4	21.5 ± 1.1

Mean ± SE, *p < 0.05. FOT, forced oscillation technique; OCT, optical coherence tomography.

### Establishment of lung disease

Changes in lung function and structure after Dox were assessed to confirm the presence of lung disease. Mechanical measurements were performed on two different mouse genotypes: wild type (++) or heterozygote (+−); for the early growth response one (Egr-1) gene, which potentially effects the severity of the response to Dox (see Methods). Lung elastance (Fig. 1B), damping (Fig. 1C) and airway resistance (Fig. 1D) were increased in the Dox group compared with the Control group. Thoracic gas volume was also increased in the Dox group compared with Control: Control/Egr-1++, 0.29 ± 0.05 mL, n = 7; Control/Egr-1+−, 0.27+− 0.02 mL, n = 8; Dox/Egr-1++, 0.38 ± 0.04 mL, n = 7; Dox/Egr-1+−, 0.35 ± 0.05 mL, n = 8; *p* < 0.05. There was no effect of genotype (Egr-1++ vs. +−) on the response to Dox. A single genotype (Egr-1+−) was therefore used for all subsequent analyses.

**Figure 2:**
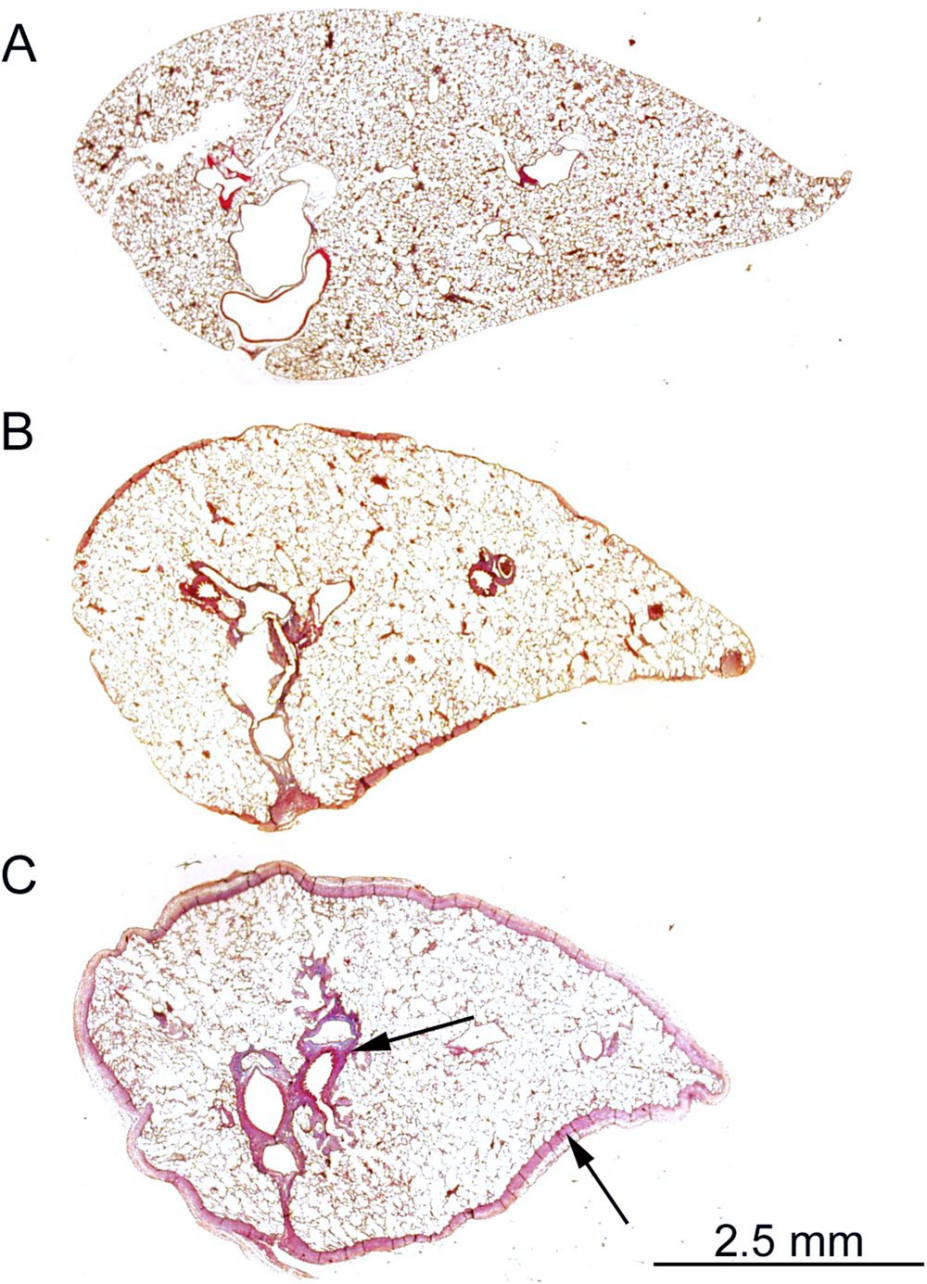
Dox produced pleural fibrosis and peribronchial fibrosis (indicated by arrow): (**A**), Control; (**B** and **C**) moderate and severe pleural fibrosis in the presence of Dox respectively.

Consistent with previous studies^16, 17^, Dox produced pleural thickening and peribronchial fibrosis (Fig. 2) with no histological evidence of lung inflammation. Semi-quantitative fibrosis score (median and range) was 1 [0–3] in the Dox group (n = 10) which was greater than the Control group (0 [0-0], n = 9, *p* < 0.001).

### OCT-based indentation of diaphragm tissue

**Figure 3:**
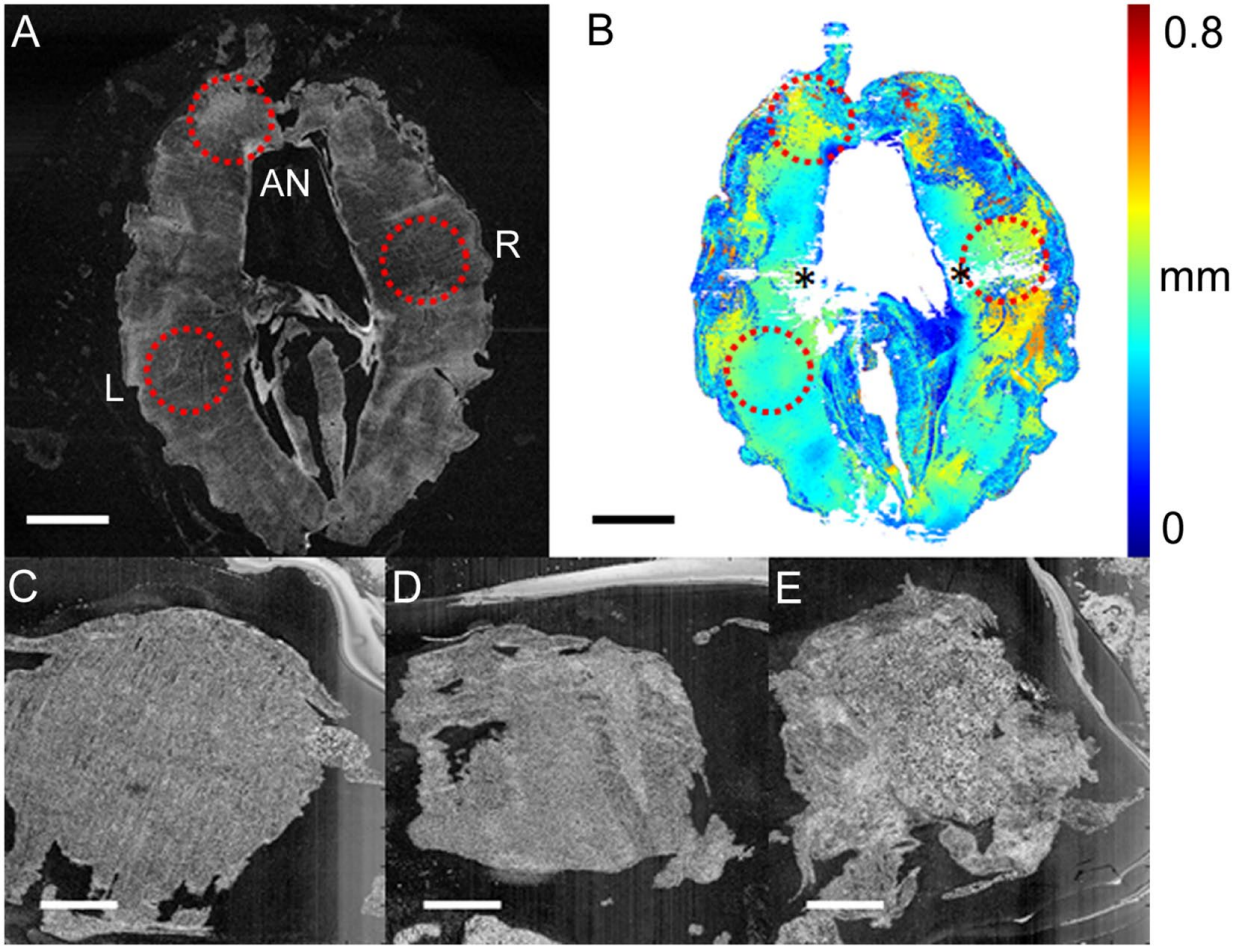
Transverse (*en face*) images of diaphragm recorded by OCT (**A**). Circles indicate the locations (left and right lateral, L and R) and anterior (AN) at which indentation was performed. Thickness map of the diaphragm (**B**); asterisks denote artefacts in thickness detection. At left and right lateral locations, normal diaphragm fibre orientation is evident in the Control group (**C**), compared with the Dox group (**D**), where connective tissue (speckled white appearance) occludes muscle. At anterior locations, diaphragm in the Control group was associated with adipose tissue, exhibiting a porous or spongy appearance (**E**). Scale bar is equal to 3 mm in (**A**) and (**B**), and 1 mm in (**C–E**).

Figure 3 shows transverse images of diaphragm tissue acquired by OCT, and includes a thickness map that was used to identify the regions for indentation (see Methods). Normal fibre orientation was apparent in diaphragms from the Control group (Fig. 3C). In comparison, a fibrotic layered appearance of the diaphragm from the Dox group (Fig. 3D) was common (6/10) compared with the Control group (0/8; chi-squared = 7.2, *p* < 0.01). Diaphragms in the Control group tended to be associated with adipose tissue (porous spongy appearance) at anterior locations (Fig. 3E). Diaphragms were less likely to be associated with adipose tissue in the Dox group (0/10) compared with the Control group (3/8, chi-squared = 4.5, *p* < 0.05).

**Figure 4:**
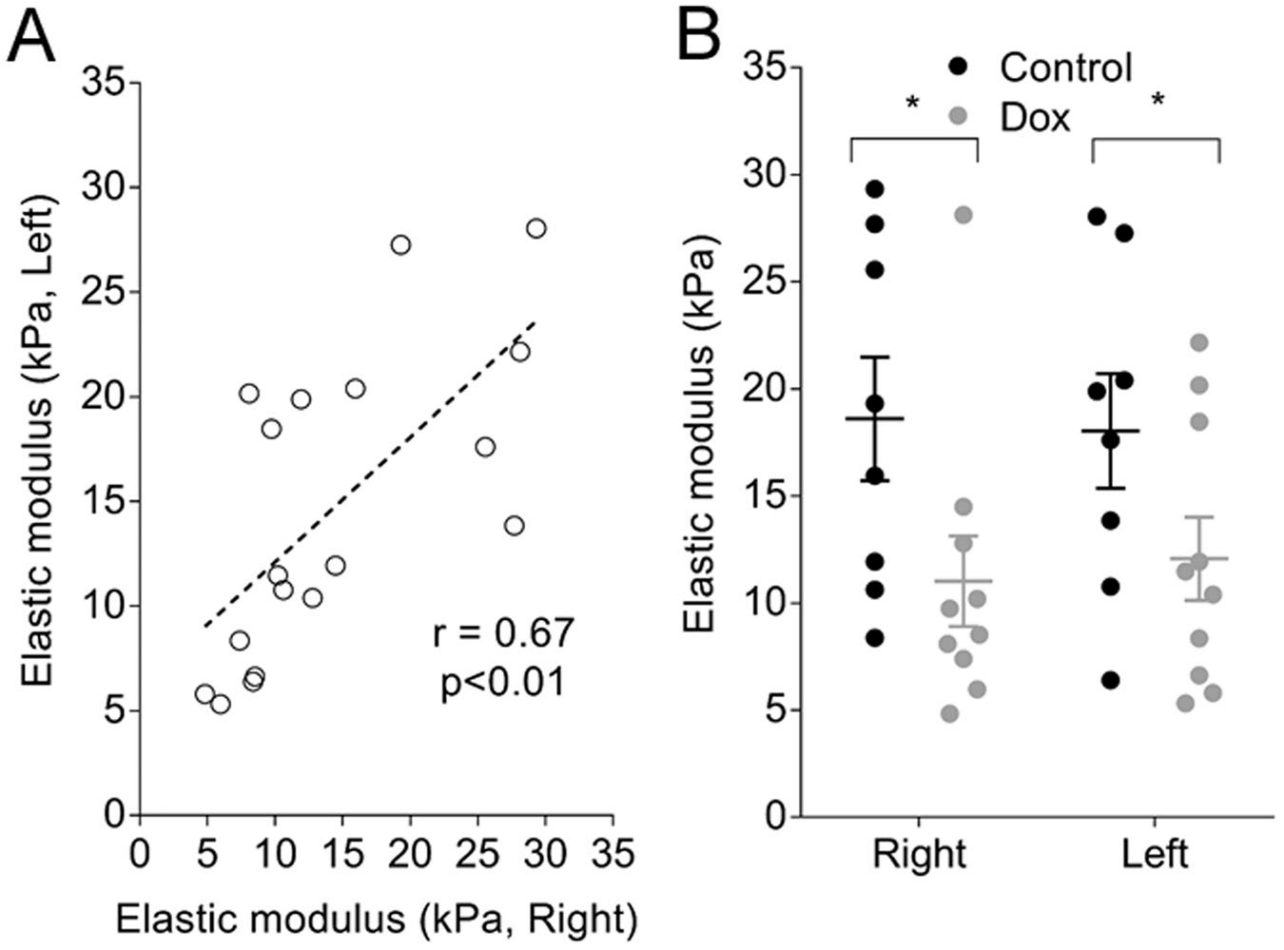
Elastic modulus of mouse diaphragm measured by OCT-based indentation. Right and left lateral measurements (data are from Control and Dox groups combined) were positively correlated (**A**, n = 18). Elastic modulus of diaphragm from the Dox group was reduced compared with the Control group (**B**). There was no effect of location. Data are mean ± SE. *p < 0.05.

Due to the potential confounding effects of soft adipose tissue in the determination of diaphragm elastic modulus, OCT-based indentation data are only reported for the left and right lateral locations. There was a correlation between the elastic modulus of the left and right diaphragm (Fig. 4A). The elastic modulus of the diaphragm from the Dox group was reduced compared with Control indicating that the diseased diaphragm was softer (Fig. 4B). There was no significant difference in the thicknesses of the diaphragm between groups: 0.68 ± 0.02 mm and 0.67 ± 0.02 mm at right and left locations respectively, in the Control group, and 0.67 ± 0.03 and 0.63 ± 0.03 mm in the Dox group.

### Organ bath assessment of diaphragmatic force

**Figure 5:**
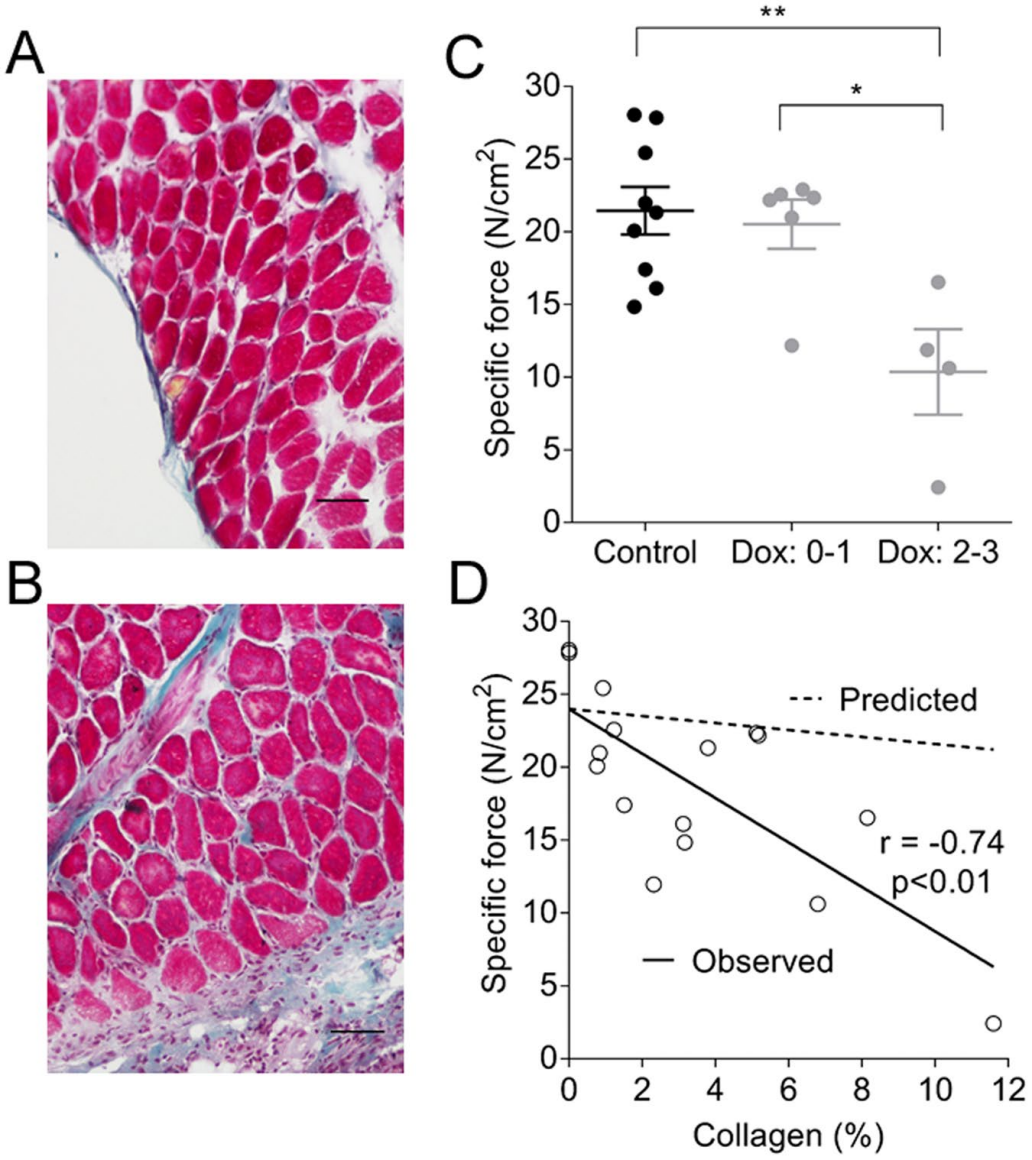
Cross sectional (scale bar = 0.1 mm) histological images of diaphragm from Control (**A**) and Dox groups (**B**). Blue collagen infiltration between muscle fibres and ligament thickening are apparent in the Dox group. Diaphragm maximum specific force (**C**) was reduced with more severe lung disease (fibrotic scores 2–3, **p < 0.01, *p < 0.01). There was an inverse correlation between diaphragm collagen (%) and force (**D**, n = 16). The predicted line shows the change in force that would be expected if muscle mass was replaced by collagen. Predicted force was calculated from the *y*-intercept of the observed line (24 N/cm^2^) multiplied by the fraction of muscle (1-%collagen/100). Data are mean ± SE.

Cross sectional histology of diaphragm tissue from Control and Dox groups is shown in Fig. 5(A and B). Thickening of the diaphragm ligament and collagen deposition was evident in the Dox group. The percentage of collagen within the diaphragm was positively correlated with lung fibrosis score from the same group of mice (n = 16, r = 0.55, *p* < 0.05). Maximum specific force in the diaphragm was reduced in Dox-treated animals with greater fibrosis score (Fig. 5C). Whilst force was reduced as the percentage of collagen within the muscle increased, the reduction in force was far greater than that predicted from a simple loss of muscle mass (Fig. 5D). There was no difference in passive diaphragmatic force or cross sectional area between groups.

## Discussion

The purpose of the study was to investigate the assessment of respiratory mechanics, specifically elastic properties of diaphragm tissue, using a new (for the respiratory system) method; OCT-based indentation. We demonstrated that OCT-based indentation clearly delineated changes in diaphragm mechanics and altered structure in a mouse model of TGF-α-induced lung disease. Below, we discuss important methodological considerations, the utility of OCT-based assessment of respiratory mechanics, and new findings on TGF-α induced respiratory disease.

The effects of TGF-α occur through activation of the epidermal growth factor receptor, which is implicated in numerous chronic respiratory diseases, including lung fibrosis, cancer, chronic obstructive pulmonary disease, asthma and cystic fibrosis^18^. In our transgenic mouse model of lung disease, TGF-α levels were increased in response to Dox. Importantly, in this model, TGF-α levels are increased in the absence of lung inflammation^16, 17^; thereby, allowing the direct effects of TGF-α to be examined. In alignment with an increasing world-wide focus on non-inflammatory mechanisms of lung disease^19^, such transgenic mouse models provide opportunities for new data on the physiological consequences of growth factors liberated as part of the disease cascade.

We initially confirmed the presence of TGF-α induced lung disease, through changes in lung mechanics (discussed further below) and the presence of pleural and peribronchial fibrosis. TGF-α effects are potentially regulated by the Egr-1 gene which is dependent upon the duration of exposure. Following extended periods of TGF-α exposure (~8 weeks on Dox), lung fibrosis is substantially enhanced in mice deficient or heterozygote for the Egr-1 gene, compared with wildtype^17^. However, we saw no differences in lung mechanics between Egr-1 genotypes (+/+ versus +/−) following a shorter 3-week exposure. For this reason, a single genotype was chosen for subsequent protocols.

Since fibrosis develops at the serosal pleura after TGF-α exposure (present study)^16, 17^, we queried whether this would also extend to parietal surfaces and infiltrate the diaphragm, thereby altering muscle mechanics. OCT-based indentation revealed a softening of the diaphragm tissue, which was accompanied by an abnormal layered appearance, consistent with fibrotic disease. While normal fibre orientation was apparent in diaphragms from control mice, this was disrupted in the Dox group. Histological sectioning revealed thickening of the diaphragm ligament in the Dox group, and increased collagen between fibres that was inversely associated with muscle force in organ bath studies. It is notable that the organ bath approach was only sufficient to detect more severe disease (Fibrosis score 1–2), in comparison with OCT-based indentation which was able to clearly delineate Control and Dox groups.

A strength of imaging-based assessment of tissue mechanics is that functional measurements are co-localised with images of structure. In the present study, OCT images revealed regional diversity between Control and Dox groups, characterised by a greater deposition of fat at anterior diaphragm locations. The presence of ‘spongy’ adipose tissue significantly reduced diaphragm elastic modulus in control animals (data not shown) and, therefore, masked real differences between Control and Dox groups. Images acquired by OCT were, therefore, used to identify regions free of adipose tissue (left and right lateral locations) so that measurements better reflected the intrinsic elastic properties of the diaphragm. Elastic modulus of diaphragm tissue at left and right locations (free of adipose tissue) was correlated (r = 0.67), suggesting that measurements were representative of tissue beyond the indenter field. To further minimise measurement error by ensuring complete and uniform contact of the indenter with the tissue, specific regions of assessment were those with the least surface heterogeneity, as determined from the thickness map.

*In vivo* translation of OCT-based indentation is a technical challenge that is dependent upon the anatomical location of the tissue of interest. With respect to the diaphragm, measurements are likely to be invasive but are feasible. In a recent study, the safety of diaphragm biopsy by laparoscopy was demonstrated without evidence of pneumothorax^20^. It is possible that OCT-based indentation could be performed under direct visualisation during laparoscopy and provide additional information than biopsy alone. Other than the diaphragm, one immediate application of OCT-based indentation is the measurement of airway elastance that may be altered in obstructive disease^21^. Elastic properties of airway passages are relatively difficult to assess in comparison with lung tissue, which dominates volume and pressure signals recorded at the mouth. Bronchoscopic delivery of OCT probes to the airway surface has been used to quantify lumen area and examine wall morphology^3, 5^. However, for assessment of airway elastance, it is necessary to control and measure the applied stress, easily achieved *in vitro* through application of a known force. *In vivo*, one solution is to integrate imaging probes with an elastic membrane of known elasticity and to subsequently calculate the applied stress from the change in membrane thickness^22^.

In an extension to OCT-based indentation, local measurements of tissue strain can be performed, referred to as compression optical coherence elastography^9, 10, 23^. By this approach, local strain is mapped across the tissue volume undergoing compression, forming mechanical contrast images (elastograms) to detect micro-mechanical features not identified by structural imaging. In the present study, compression elastography was performed alongside OCT-based indentation. An important distinction to OCT-based indentation is that compression elastography, in its simplest form, only provides measurement of strain, which in the absence of known local stress, is a relative measure of the mechanical properties. Strain has been used to identify tissue pathology via textural features^24^; however, in this study, there was no significant change in mechanical texture between animal groups, suggesting that the scale at which the mechanical properties vary in the disease model is lower than the elastogram resolution (~10–100 µm). Assessment based solely on local strain is somewhat limited and would be greatly advanced if local stress could also be determined. Recently, aided by the aforementioned elastic membrane to measure local stress, images of absolute local elastance (local stress/local strain) were achieved with elastography in breast tissue^25^. A combination of indentation loading with local quantification of elastance via OCT elastography may prove to be the most useful approach to assess mechanical properties in respiratory science.

Due to technical constraints, it was not possible to directly correlate data generated by OCT-based indentation with organ bath assessment; instead, the organ bath approach was employed to provide an initial indication that the diaphragm was affected in the mouse model of lung disease, before testing a new methodology. Moreover, data generated from OCT-based indentation provides different and complementary information. OCT-based indentation reflects the mechanical properties of the tissue in the orientation perpendicular to the muscle fibres (as would be the case *in vivo*, analogous to a biopsy procedure); whereas, the organ bath approach assesses muscle mechanics in parallel to the contractile fibres. Together, these data provide some insight as to how diaphragm function may be altered in TGF-α induced lung disease. We propose that softening of the diaphragm tissue observed by OCT-based indentation occurs due to decoupling of skeletal muscle fibres by fibrotic infiltration, resulting in an energy-inefficient contractile process that attenuates the production and transmission of force throughout the diaphragm tissue.

There are several other less likely mechanisms that may lead to a reduction in diaphragmatic force after TGF-α exposure. There is evidence that TGF-α promotes muscle wastage of distal hind limbs in mice^26^. A decrease in muscle bulk cannot explain our findings since diaphragmatic force was normalised to muscle cross-sectional area. Changes in diaphragmatic force may be a direct consequence of collagen deposition. Intra-diaphragmatic collagen has been documented in patients with chronic obstructive pulmonary disease^27^, and may increase the work of breathing. An increased proportion of collagen within the muscle layer reduces the number of functional contractile units in a given cross sectional area of muscle. However, decreases in diaphragm force were far greater than expected from a simple change in muscle composition (Fig. 5D).

Additional studies are required to determine if there is a consistent decrease in diaphragmatic adipose tissue in the current mouse model of TGF-α lung disease. Other studies have reported decreases in body weight^16, 17^ and absolute fat content following TGF-α exposure^26^. There is also evidence that mesenchymal progenitor cells are able to differentiate into both collagen type-I producing cells and adipocytes^28^. We can only speculate that a TGF-α driven fibrotic process may be associated with a down regulation of differentiation of adipocyte cells.

The present study provides further mechanistic insight into changes in lung mechanics that occur in TGF-α induced lung disease. Previous studies reported changes in total respiratory compliance and resistance^16, 17^, whereas, our study isolated these changes to lung elastance and damping, airway (Newtonian) resistance and lung volume. In the absence of pulmonary fibrosis (fibrosis was not present in alveolar walls), increased lung elastance can be attributed to pleural thickening and greater elastic load. Tissue damping on the other hand reflects viscous properties of the lung which will be affected by fibrosis in the peribronchial space, as well as pleural fibrosis. Peribronchial fibrosis may also account for increased airway resistance, likely as a result of airway-parenchymal decoupling^29^, promoting airway collapse and increasing gas volume determined by plethysmography at atmospheric elastic-equilibrium lung volume.

Finally, taken together, findings on lung tissue and diaphragm have clinical significance. In human disease, a variety of methods are used to assess chest wall disease in those with varying degrees of breathlessness, often in relation to exposures to agents such as asbestos. Often the degree of symptoms, such as breathlessness is out of proportion with functional and radiological changes. It has been suggested that pleural thickening involving the diaphragm has a greater effect on breathlessness than thickening elsewhere on the chest wall^30^. The present study suggests that changes within the diaphragm not observable on routine imaging may still alter diaphragm function and contribute to symptoms.

In conclusion, the present study used OCT-based indentation to demonstrate impaired diaphragm mechanics following TGF-α exposure, which may contribute to morbidity and mortality in chronic lung disease. We propose that assessment of tissue mechanics by OCT has diagnostic potential across a diverse range of chronic lung diseases.

## Methods

Our experimental approach was to establish TGF-α-induced lung disease in transgenic mice and to quantitatively measure these changes using established functional and histological methodologies, notably the FOT and plethysmography, and *in vitro* organ bath experiments on excised diaphragm tissue. The utility of OCT-based indentation was then investigated through the assessment of diaphragm tissue structure and mechanics from mice with and without TGF-α-induced lung disease.

### Animal handling

The mouse model used is described fully in a previous publication^17^, which in this study was re-derived on a BALB/c background (The Jackson Laboratory). Mice bi-transgenic for Clara cell secretory protein-rtTA(+/−)/[tetO](7)-TGF-α(+/−) and wild type (++) or heterozygote (+−) for the Egr-1 gene, conditionally expressing TGF-α in the lungs in response to Dox were used in this study. Young mice (4–5 weeks) were fed Dox or a control diet (Control) for three weeks before being randomly allocated to the *in vivo* or *in vitro* protocol. Mice were anaesthetised by intraperitoneal injection of xylazine (2 mg/mL) and ketamine (40 mg/mL) at a dose 0.1 mL/10 g body weight for *in vivo* lung function, or overdosed with the same solution (0.2 mL/10 g). All experiments were approved by the Animal Ethics Committee at Telethon Kids Institute. All methods were performed in accordance with the Australian Code of Practice for the Care and Use of Animals for Scientific Purposes (National Health and Medical Research Council, Australia, 7^th^ Edition).

### Forced oscillation technique and plethysmography

Lung mechanics were assessed in anaesthetised and mechanically ventilated mice by low frequency FOT^31^. Outcome variables included airway resistance, lung elastance and damping. Plethysmography was used to measure thoracic gas volume at atmospheric elastic-equilibrium lung volume^32^. The trachea was occluded at end expiration and the intercostal muscles were stimulated with intramuscular electrodes to induce inspiratory efforts. Associated changes in tracheal pressure and plethysmograph box pressure were used to calculate volume according to Boyle’s law.

### Organ bath studies

Longitudinal strips (2–3 mm wide) of intact muscle fibres were dissected from the right hemi-diaphragm and mounted in an *in vitro* muscle test system (1200A, Aurora Scientific, Canada) containing mammalian Ringer solution (pH 7.3) bubbled with carbogen at 25 °C^33^. Diaphragm strips were stimulated at optimal muscle length (L_o_) with 0.2 ms supramaximal square wave pulses at 80 Hz to determine maximum tetanic force (g). Passive force development was determined by passive lengthening from 0.8 L_o_–1.1 L_o_ at 0.05 L_o_/s. Force (g) was normalised to cross-sectional area (N/cm^2^) calculated from muscle fibre length, muscle mass and density (1.056 g/cm^3^).

### OCT-based indentation

Whole diaphragms were imaged (Fig. 3A) using a benchtop spectral-domain OCT system^34^. OCT captured volumetric images of tissue microstructure, at an axial and lateral resolution of 8 μm and 11 μm, respectively^34^. Indentation was performed at left and right lateral, and anterior locations (Fig. 3A). The diaphragm muscle was compressed by a 3-mm diameter, flat, cylindrical indenter (a weighted rod), which applied a physiologically relevant stress onto the sample, i.e., 0.92 kPa or ~10 cmH_2_O. The imaging beam was incident on the sample (typically 500 mm thick) from the opposite side with respect to the indenter. The stress at the tissue surface was determined from the known weight placed on the indenter and the OCT-measured surface area of tissue in contact with the indenter. The strain of the diaphragm was captured using OCT by measuring its thickness in the uncompressed and compressed states. To reduce artefacts associated with tissue inhomogeneity, flat regions of the diaphragm were chosen for assessment, qualitatively, aided by a thickness map (Fig. 3B), generated from OCT volumes using a Canny edge detector. OCT-based indentation was performed on the same regions (to within 1 mm) in all diaphragms; these regions were chosen prior to the measurements presented in this paper.

At each location, the elastic (Young’s) modulus of the muscle was calculated as the ratio of the stress applied to the tissue over the strain (∆thickness/initial thickness), i.e., Hooke’s Law. This model was employed, in contrast to common indentation models, as the diaphragm thickness (~500 μm) was significantly smaller than the indenter diameter (3 mm). A single value of elastic modulus was obtained by determining the average strain in the region under test and relating this to the measured stress. The error in the measured elastic modulus (derivation in Supplement 1), in an ideal case, can be calculated by propagating the error associated with the measured parameters, *i.e*., the mass, the diameter and the displacement of the indenter, and the thickness of the sample. The error, given that the elastic modulus of the tissue is 14 kPa (combined average of Control and Dox groups), was measured as 0.83 kPa (standard deviation). Furthermore, biological tissue is typically non-linear elastic, exhibiting a higher effective elastic modulus with increased applied stress. The non-linearity was measured by applying a higher stress of 2.4 kPa to all diaphragms; at 2.4 kPa the average elastic modulus was 6.5% greater than at 0.92 kPa. The non-linearity should be taken into account when comparing diaphragm elastic modulus measured under different stresses.

### Tissue analyses and histology

After the removal of the diaphragm for *in vitro* studies, lungs were fixed by formalin immersion and embedded longitudinally to acquire cross sections stained by Masson’s Trichrome. Fibrosis was assessed semi-quantitatively by a blinded observer (L.B): 0 (none); 1 (mild); 2 (moderate) and 3 (severe). Diaphragm strips (16/19) were embedded in tragacanth and frozen in isopentane cooled in liquid nitrogen. Cross sections were also stained with Masson’s Trichrome to measure collagen as a percentage of cross sectional area. In some animals (Egr-1+−), concentration of TGF-α in lung homogenates was assessed by enzyme-linked immunosorbent assay (Elabscience, E-EL-H1586).

### Analysis and statistics

Data were transformed where necessary to ensure the assumptions of normality and homoscedasticity of variances for the parametric tests were satisfied. Where this was not possible equivalent non-parametric tests were used. Linear associations were assessed by correlation analysis (Pearson or Spearman based on normality). All statistics were performed by GraphPad Prism (GraphPad Software, version 7.02).

## Electronic supplementary material

Derivation of the error in the measured elastic modulus
