# Supplementary material for: Optical coherence tomography-based contact indentation for diaphragm mechanics in a mouse model of transforming growth factor alpha induced lung disease
Source: Sci Rep. 2017 May 4;7:1517. doi: 10.1038/s41598-017-01431-x (PMC5431417; doi:10.1038/s41598-017-01431-x)
Supplement: Supplementary file 1 — Derivation of the error in the measured elastic modulus [file 41598_2017_1431_MOESM1_ESM.pdf]

Optical coherence tomography-based contact indentation for diaphragm mechanics in a mouse model of transforming growth factor alpha induced lung disease

Kimberley C. W. Wang[1,*], Chrissie J. Astell[2], Philip Wijesinghe[3,4], Alexander N. Larcombe[1,5], Gavin J. Pinniger[2], Graeme R. Zosky[1,6], Brendan F. Kennedy[4,7], Luke J. Berry[1], David D. Sampson[3,8], Alan L. James[9], Timothy D. Le Cras[10], Peter B. Noble[1,2,11]

[1]Telethon Kids Institute, The University of Western Australia, Subiaco, Western Australia, Australia; [2]School of Anatomy, Physiology and Human Biology, The University of Western Australia, Perth, Western Australia, Australia; [3]Optical+Biomedical Engineering Laboratory, School of Electrical, Electronic & Computer Engineering, The University of Western Australia, Perth, Western Australia, Australia; [4]BRITElab, Harry Perkins Institute of Medical Research, QEII Medical Centre, Crawley, Western Australia, Australia; [5]School of Public Health, Curtin University, Perth, Western Australia, Australia; [6]University of Tasmania, Hobart, Tasmania, Australia; [7]School of Electrical, Electronic & Computer Engineering, The University of Western Australia, Perth, Western Australia, Australia; [8]Centre for Microscopy, Characterisation & Analysis, The University of Western Australia, Perth, Western Australia, Australia; [9]Sir Charles Gairdner Hospital, Nedlands, Western Australia, Australia; [10]Cincinnati Children's Hospital Medical Center, Cincinnati, Ohio, USA; [11]Centre for Neonatal Research and Education, School of Paediatrics and Child Health, The University of Western Australia, Perth, Western Australia, Australia.

Correspondence should be addressed to:
Kimberley CW Wang, PhD.
Telethon Kids Institute
PO Box 855
West Perth, WA 6872
Australia
Email: kimberley.wang@telethonkids.org.au

Supplement 1

Uniaxial compression and indentation testing is commonly used in characterizing the Young's modulus of materials[1]. Due to their simplicity, the accuracy of the estimated modulus is directly related to the accuracy of the measured force and displacement, and the tolerances of their components. The error associated with these parameters may be quantified and readily propagated through the mechanical model, deriving the expected error in the estimate of Young's modulus; and we do so for the method presented in this paper.

Under the assumption of uniaxial compression of the sample, Young's modulus is defined as $E = \sigma/\varepsilon$, where stress is $\sigma = F/A$, and strain is $\varepsilon = u/L$; F is the force, A is the surface area of the indenter, and u is the displacement of the indenter during sample compression. This assumption is reasonable when the sample thickness, $L \approx 0.66$ mm, is a few times smaller than the diameter of the flat indenter, $\alpha = 3$ mm. In the method presented in this paper, the error in the estimated Young's modulus, in an ideal case, is a product of the error in measuring the mass (m), the diameter (α) and the displacement (u) of the indenter, and the thickness (L) of the sample. The corresponding errors (as one standard deviation) for m, α, u and L are $\delta m = 0.5$ mg (accuracy of the scale), $\delta\alpha = 0.05$ mm (tolerance of fabrication), $\delta L = 1.2$ μm and $\delta u = 1.2$ μm (accuracy of OCT). u and L were taken as the average values across all experiments: $\bar{u} = 0.040$ mm and $\bar{L} = 0.66$ mm.

Given these assumptions, the systematic error (δ) can be estimated as follows:

$$F = mg = 6.14\times10^{-4}\cdot9.81 = 6.02\times10^{-3}\ [\mathrm{N}] \quad (1)$$
$$\delta F = \delta m\cdot|g| = 0.5\times10^{-6}\cdot9.81 = 4.9\times10^{-6}\ [\mathrm{N}] \quad (2)$$
$$A = \pi(\alpha/2)^2 = \pi(1.5\times10^{-3})^2 = 7.1\times10^{-6}\ [\mathrm{m}^2] \quad (3)$$
$$\delta A = |A|\cdot|\pi|\cdot2\cdot(\delta\alpha/|\alpha|\cdot1/4) = 7.1\times10^{-6}\cdot\pi\cdot(0.05/3)/2 = 1.9\times10^{-7}[\mathrm{m}^2] \quad (4)$$
$$\sigma = F/A = 6.02\times10^{-3}/7.1\times10^{-6} = 850\ [\mathrm{Pa}] \quad (5)$$
$$\delta\sigma = |\sigma|\cdot(\delta F/|F| + \delta A/|A|) = 850\cdot(4.9\times10^{-6}/6.02\times10^{-3} + 1.9\times10^{-7}/7.1\times10^{-6}) = 23\ [\mathrm{Pa}] \quad (6)$$
$$\varepsilon = \bar{u}/\bar{L} = 0.040/0.66 = 6.1\times10^{-2} \quad (7)$$
$$\delta\varepsilon = |\varepsilon|\cdot(\delta\bar{u}/|\bar{u}| + \delta\bar{L}/|\bar{L}|) = 6.1\times10^{-2}\cdot(1.2\times10^{-3}/0.040+1.2\times10^{-3}/0.66) = 1.9\times10^{-3} \quad (8)$$
$$E = \sigma/\varepsilon = 850/6.1\times10^{-2} = 14\times10^{3}\ [\mathrm{Pa}] \quad (9)$$
$$\delta E = |E|\cdot(\delta\sigma/|\sigma| + \delta\varepsilon/|\varepsilon|) = 14\times10^{3}\cdot(23/850+1.9\times10^{-3}/6.1\times10^{-2}) = 8.3\times10^{2}\ [\mathrm{Pa}] \quad (10)$$

Therefore, given the Young's modulus of the sample is 14 kPa, the error is 0.83 kPa. Interestingly, the greatest contributions to the error come from the OCT-based detection of displacement and the tolerance of the indenter diameter. Both parameters can be improved upon, particularly in OCT, where the estimation of displacement may be obtained directly from the interferometric signal with sub-micrometer accuracy[2].

References

1 Czichos, H., Saito, T. & Smith, L. *Springer handbook of materials measurement methods*. Vol. 978, (Springer Berlin, 2006).

2 Schnell, U., Gray, S. & Dändliker, R. Dispersive white-light interferometry for absolute distance measurement with dielectric multilayer systems on the target. *Optics Letters* **21**, 528-530 (1996).
